# The impact of fall history on instability, disability, and anxiety in patients with chronic neck pain

**DOI:** 10.55730/1300-0144.6149

**Published:** 2025-12-21

**Authors:** Bülent BOZYİĞİT, Emre SÖYLEMEZ, Aydın Sinan APAYDIN

**Affiliations:** 1 Department of Neurosurgery, Private Sağlık Hospital, İzmir, Turkiye; 2 Department of Audiometry, Vocational School of Health Services, Karabük University, Karabük, Turkiye; 3 Department of Neurosurgery, Faculty of Medicine, Karabük University, Karabük, Turkiye

**Keywords:** Chronic neck pain, fall history, postural stability, anxiety, balance assessment

## Abstract

**Background/aim:**

This study aimed to investigate the impact of fall history on postural stability, disability, and anxiety in individuals with chronic neck pain (CNP). It was hypothesized that patients with a history of falls would exhibit greater postural instability, perceived disability, and anxiety levels compared with those without a fall history and healthy controls.

**Materials and methods:**

A total of 62 participants were included: 16 CNP patients with a history of falling (Group Ia), 21 CNP patients without falls (Group Ib), and 25 healthy controls. Assessments included the Neck Disability Index (NDI), Beck Anxiety Inventory (BAI), and Visual Analog Scale (VAS) for instability, and static postural stability was measured using a force platform under four sensory conditions.

**Results:**

Group Ia demonstrated significantly higher NDI, BAI, and VAS scores than Group Ib and controls (p < 0.05). Postural stability was significantly worse in Group Ia under eyes-closed conditions on both firm and foam surfaces (p < 0.05). Group Ib showed balance deficits only on the foam surface with eyes closed. No significant differences were found under eyes-open conditions (p > 0.05).

**Conclusion:**

Fall history in patients with chronic neck pain is associated with greater balance impairments and increased anxiety, suggesting that a simple self-reported fall history may serve as a low-cost indicator of disrupted postural control. These findings may provide a practical guide for early screening of fall risk in clinical settings and highlight the importance of implementing preventive strategies for individuals with chronic neck pain.

## 1. Introduction

Neck pain affects between 30% and 50% of the adult population and can have detrimental effects on overall health and quality of life [1,2]. Despite its ubiquity, most cases of chronic neck pain lack a clear underlying diagnosis, leaving patients undiagnosed for extended periods [3]. This diagnostic ambiguity often leads to suboptimal treatment strategies, prolonged disability, and increased psychological distress, including heightened levels of anxiety and depression. In the absence of a definitive medical explanation, patients may experience frustration and uncertainty, which can further exacerbate their pain perception and reduce engagement with rehabilitation efforts. Additionally, chronic neck pain has been linked to reduced participation in daily activities and social withdrawal, ultimately diminishing overall quality of life and increasing the burden on healthcare systems.

The cervical spine maintains a complex and indirect relationship with the visual, vestibular, and motor systems [1]. Emerging evidence indicates that chronic neck pain may interfere with sensorimotor control and proprioception, potentially contributing to balance disturbances and increased fall risk in certain individuals [1]. However, findings across studies remain inconsistent, and it is still unclear whether these functional impairments result directly from chronic pain, represent compensatory adaptations, or are influenced by coexisting conditions [4–6]. Muscle spindles are particularly abundant in the upper cervical spine and paraspinal muscles, especially in the deep suboccipital region [7]; therefore, it is believed that pathologies in the upper cervical region are more likely to impair balance and postural control. A more refined understanding of the interplay among chronic neck pain, sensorimotor function, and psychological well-being is, therefore, crucial for developing more effective assessment and treatment strategies.

Falls, especially among older adults, represent a significant public health concern, adversely affecting quality of life not only through physical injuries but also via psychological and social consequences [8]. Fear of falling can restrict individuals’ mobility, leading to physical functional decline, social isolation, and depression [9]. Impairments in the balance system do not solely cause falls; multiple factors—including muscle weakness, neuromuscular coordination deficits, medication use, visual problems, and cognitive factors—collectively increase the risk of falling. Therefore, fall risk assessment requires a multidimensional, comprehensive approach, and it is also crucial to separately evaluate the psychosocial and physical impacts of both falls and fear of falling.

Previous studies in the literature have primarily focused on the effects of chronic neck pain (CNP), its severity, or its duration on balance, disability, and anxiety [10–12]. However, to our knowledge, no study has specifically examined how fall history influences these factors in individuals with CNP. Therefore, this study aims to investigate the impact of fall history on balance, disability, and anxiety in patients with chronic neck pain by comparing those with and without a history of falls, and with healthy individuals. We hypothesize that patients with a greater history of falls are more prone to increased instability, perceived disability, and higher levels of anxiety.

## 2. Methods

### 2.1. Study design and participants

Written informed consent was obtained from all participants. In addition, ethical approval was obtained from the noninterventional ethics committee of the relevant university for the research (date: April 16, 2025; decision no: 2025/2210).

This study included 39 patients who presented to the neurosurgery outpatient clinic with CNP (>3 months). The medical history and fall status in the past year were questioned. A fall was defined according to the World Health Organization definition as an event which results in a person coming to rest inadvertently on the ground, floor, or other lower level.^1^ Sixteen CNP individuals with a history of falling were included in Group Ia. Twenty-one CNP individuals without a history of falling were included in Group Ib. Twenty-five individuals with similar age and gender characteristics, with no history of falls or neck pain, and who had never undergone any prior clinical evaluation or treatment for neck pain were included in the control group. A specialist neurosurgeon performed a clinical evaluation of these patients, and the patients were administered the Neck Disability Index (NDI), the visual analog scale (VAS), and the Beck Anxiety Inventory (BAI). Postural stability was evaluated using a force plate.

Inclusion criteria for the study groups were as follows: individuals aged 18–65 years; complaints of CNP for more than 3 months; admission to a neurosurgery outpatient clinic; and clinical evaluation by a specialist neurosurgeon. For Group Ia, participants had a history of falling in the past year, while Group Ib included individuals without a history of falling during the same period. Inclusion criteria for the control group were the absence of neck pain and similar age and gender characteristics to the CNP groups. Exclusion criteria for all participants included a history of psychiatric illness (such as depression or other mood disorders), previous episodes of vertigo, or musculoskeletal system disorders, use of medications that may affect balance (such as sedative drugs), and neurological disorders.

### 2.2. Outcome measures

#### 2.2.1. BAI

Anxiety levels of participants were evaluated using the BAI, consisting of 21 items. The Turkish adaptation and validation were conducted by Ulusoy et al [13]. Each item is rated on a four-point Likert scale (0–3), and the total score ranges from 0 to 63. Higher scores indicate higher levels of anxiety.

#### 2.2.2. NDI

The Turkish adaptation of the NDI was conducted by Kesiktaş et al [14]. This 10-item index assesses functional limitations related to neck pain. Each item is scored between 0 and 5, with higher scores indicating greater disability.

#### 2.2.3. VAS

Participants’ instability levels were evaluated using the VAS. Participants were presented with a 10-cm line ranging from “0 = no instability” to “10 = extreme instability” and asked to mark the point that best represented their perceived instability. The distance from the start of the line was measured in centimeters to determine the score.

#### 2.2.4. Postural stability assessment

Static balance skills were assessed using the Center of Pressure (COP) parameter via a force platform (Force Plate, Bertec Corporation, Ohio, USA). The Limit of Stability (LOS), representing the individual’s balance boundary, was first determined by asking participants to move their bodies maximally forward, backward, and side to side. COP sway was then evaluated under four conditions: Eyes open–firm surface, eyes closed–firm surface, eyes open–foam surface, eyes closed–foam surface. Participants were instructed to remove their shoes, step onto the platform, stand with their feet together, and maintain a still posture for 10 s in each condition. The device calculated a stability score by expressing COP sway as a percentage relative to LOS, using the following formula: Stability Score = (LOS-TotalSwayArea)/LOS× 100.

### 2.3. Statistical analysis

A post hoc power analysis was performed using the “eyes closed–foam surface” condition, which showed the largest difference among the three groups. The effect size (Cohen’s f) was 0.46, and the statistical power of the study was calculated as 89% at the α = 0.05 level.

Statistical analyses were performed using IBM SPSS Statistics 21.0 (IBM Corp., Armonk, NY, USA). Normality was tested using the Shapiro–Wilk test. Normally distributed variables were reported as mean ± standard deviation, while nonnormal variables were reported as median (min–max). One-way ANOVA was used to compare three groups when normality and homogeneity of variances were satisfied; otherwise, the Kruskal–Wallis test was used. Bonferroni correction was applied for multiple comparisons.

## 3. Results

No significant differences were observed between groups in age, sex, or comorbidities (p > 0.05). There was no significant difference in neck pain duration between Group Ia and Group Ib (p = 0.438). However, the NDI score was significantly higher in Group Ia compared with Group Ib (p = 0.002). Demographic characteristics, neck pain duration, NDI scores, and comorbidities by group are presented in Table 1.

Group Ia had significantly higher BAI scores compared with both Group Ib and the control group; Group Ib also had higher BAI scores than the control group (p < 0.05). The VAS score of Group Ia was significantly higher than those of Group Ib and the control group (p < 0.05), but the difference between Group Ib and the control group was not statistically significant (p = 0.058). BAI and VAS scores by group are shown in Figure.

There were no significant differences in postural stability on firm and foam surfaces with eyes open (p > 0.05). However, Group Ia showed significantly worse postural stability than the control group on the firm surface with eyes closed (p = 0.046). Additionally, Group Ia exhibited significantly worse postural stability on the foam surface with eyes closed compared with both Group Ib and the control group (p < 0.05). Postural stability scores by group are presented in Table 2.

## 4. Discussion

Proprioceptive input is one of the essential sensory modalities required for maintaining balance. Cervical spine problems are thought to impair proprioceptive input from the cervical region, potentially leading to cervicogenic dizziness or balance disorders. Such impairments increase the risk of falls, which are known to be significant causes of morbidity and mortality. Moreover, even when falls do not result in serious physical injury, they can still lead to a considerable fear of falling, psychological distress, and functional limitations. This study aimed to investigate the effects of falls in individuals with CNP, a factor known to disrupt balance. Specifically, we compared balance, anxiety levels, and neck disability between CNP patients with and without a history of falls. Our findings provide supporting evidence that CNP negatively affects static balance and anxiety levels. Furthermore, as demonstrated in our study, patients in Group Ia (those prone to falls) had significantly higher NDI and VAS scores than those in Group Ib (those without a history of falls).

Alshehri et al. [12] investigated the relationship between cervical muscle strength, functional balance, and limits of stability in individuals with nonspecific CNP. The authors emphasized that cervical muscle strength is an important parameter for maintaining balance and reported a negative association between neck pain and balance performance. Similarly, Apaydin and Soylemez [5] found that individuals with nonspecific CNP did not exhibit impairments in single-task walking compared with asymptomatic controls; however, their dual-task walking performance was significantly affected. Another study [4] evaluated cervicocephalic kinesthetic sense (i.e., cervical proprioceptive input) and postural balance in individuals with nontraumatic CNP. The authors observed only limited kinesthetic impairment in these patients and did not detect any major deficits in overall balance. Discrepancies among these studies may be attributed to differences in neck pain severity, as suggested by Alshehri et al. [12]. In line with this, our findings revealed that CNP patients with a history of falling exhibited higher NDI scores and poorer postural stability. Another possible explanation may involve interindividual variability, particularly in the extent to which cervical proprioceptive input is impaired. As is well known, the muscles at the C1–C3 level—especially the suboccipital muscles—contain a high density of muscle spindles [7]. Therefore, the localization of structural or functional impairments in the cervical region may play a critical role in the development of postural instability.

Our study demonstrated that individuals with CNP but without a history of falls exhibited impaired postural stability only under altered sensory conditions, such as standing on a soft surface. In contrast, those with a history of falls showed significant balance impairments on both firm and soft surfaces. Taken together, these findings suggest that a previous history of falls may serve as a simple clinical indicator of impaired postural stability in individuals with chronic neck pain. This association is also supported by previous studies conducted in the general population [15].

Although balance can be assessed using simple functional tests such as the Romberg or Timed Up and Go tests, these tools generally have low sensitivity and specificity. Therefore, more objective and reliable assessments often require advanced technologies such as static or dynamic posturography using force platforms. However, these systems are costly and not readily accessible in most clinical settings. In this context, inquiring about a history of falls in individuals with CNP may offer a practical and low-cost means of predicting impaired postural stability. Moreover, the literature reports that even a single fall is one of the strongest predictors of future falls [16].

Clinically, reducing the risk of falls should be considered an additional goal of care alongside alleviating chronic neck pain, as this may help prevent future complications associated with postural instability, including falls or other related injuries. Furthermore, prolonged postural instability may contribute to the development of maladaptive postural patterns [17], which have been associated with alterations in spinal segmental alignment and spinal biomechanics [18,19]. Early intervention is essential to prevent further deterioration, particularly considering that most individuals with chronic neck pain belong to the older population [20]. Accordingly, our results indicate that the evaluation of chronic neck pain must extend beyond mechanical, inflammatory, or malignant causes and should also address the patient’s functional status and quality-of-life improvements. Therefore, besides conventional rehabilitation protocols, which prioritize pain reduction and range of motion restoration, this approach may prove unfruitful in patients with balance issues. These patients may need additional exercises, such as global postural reeducation, cervical repositioning exercises, balance training on uneven surfaces, and visual–motor coordination drills. In this context, Mendes Fernandes et al. [21] emphasized that global postural reeducation and specific exercise interventions were effective in improving neck mobility and reducing disability in this patient group. However, no statistically significant improvements in postural alignment were observed, highlighting the need for more intensive sensorimotor training protocols to enhance postural control.

Another underappreciated aspect of chronic neck pain is that fear and anxiety are associated with concerns about future falls and accidents. As revealed by our results, BAI scores were significantly elevated in fall-prone patients. This heightened anxiety may lead to further avoidance of physical activity and therapeutic exercise, both of which are crucial for improving postural stability and neuromuscular control in this population. Moreover, anxiety can cause patients to adopt compensatory or maladaptive postural strategies—such as increased cocontraction, rigidity, or guarded movements—which may paradoxically exacerbate instability. This creates a vicious cycle: fear of falling leads to altered motor behavior, which disrupts normal sensorimotor integration and ultimately increases the risk of falling. A clear clinical example of this maladaptive loop is Persistent Postural-Perceptual Dizziness (PPPD) [22]. PPPD is a chronic functional vestibular disorder characterized by nonspinning dizziness, unsteadiness, and hypersensitivity to motion cues, often triggered or maintained by anxiety and altered postural control strategies. Patients with PPPD frequently rely on visual and somatosensory cues excessively and inflexibly, while underutilizing vestibular input, resulting in poor adaptability and heightened postural threat perception. In individuals with chronic neck pain, particularly those with comorbid anxiety, similar mechanisms may be at play—leading to sensorimotor mismatch, overreliance on vision, and stiffening behaviors that interfere with normal balance strategies. Therefore, addressing psychological components such as fear and anxiety is not only beneficial for pain management but also essential for restoring healthy postural control.

Despite its novel approach, the present study has several limitations that should be acknowledged. First, the cross-sectional design precludes causal inference about the observed relationships among postural stability, neck pain, and fall-related factors. Second, the relatively small sample size and the predominance of female participants across all groups may limit the generalizability of the findings. In addition, the absence of objective assessments of the oculomotor and vestibular systems restricts a more comprehensive evaluation of balance control mechanisms. The lack of data on the number and frequency of falls limited a more detailed analysis of fall-related risk. Furthermore, physical activity level and body mass index, both known to influence postural control and fall risk, were not assessed in this study. These factors should be taken into consideration when interpreting the results. Future studies employing longitudinal designs, larger and more diverse samples, and comprehensive assessments including fall frequency, objective oculomotor and vestibular measures, physical activity level, and body mass index may better elucidate the underlying mechanisms and strengthen the clinical relevance of the findings.

## 5. Conclusion

Our study demonstrated that individuals with CNP experience increased postural instability, reduced functional capacity, and impaired psychological well-being. Moreover, a history of falls in these individuals further exacerbates balance impairments and anxiety levels. In addition, greater neck disability was associated with an increased risk of falling. Overall, these findings underscore the importance of evaluating fall risk and balance-related factors in individuals with CNP to help prevent future disability and may provide a practical framework for the early screening of fall risk in this population.

## Figures and Tables

**Figure:**
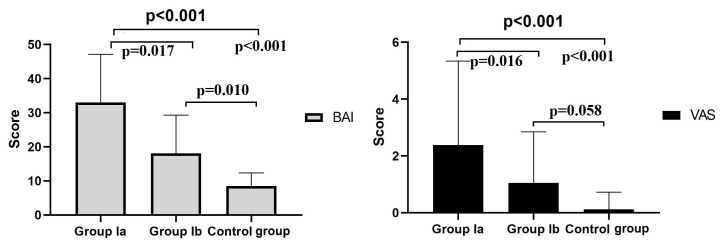
Comparison of the groups according to BAI and VAS scores.

**Table 1:** Demographic characteristics, comorbidities, neck pain duration, and NDI scores by group.

	Group Ia (n = 16)	Group Ib (n = 21)	Control group (n = 25)	p
**Age (years)**, mean ± sd.	36.87 ± 12.82	33.23 ± 9.24	33.12 ± 8.10	0.439^a^
**Sex, (n)**				0.237^c^
Female	14 (87.5%)	16 (76.2%)	16 (64.0%)	
Male	2 (12.5%)	5 (23.8%)	9 (36.0%)	
**Neck pain duration (months)**, median (min–max) (Q1–Q3)	48 (3–180) [15–60]	24 (2–96) [12–48]	-	0.438^b^
**NDI score**, median (min–max) (Q1–Q3)	30 (22–26) [22–34.5]	22 (6–33) [13–26]	**-**	**0.002** b
Comorbidities (n)				
Hypertension	8 (50.0%)	9 (42.9%)	8 (32.0%)	0.497^c^
Diabetes mellitus	8 (50.0%)	11 (52.4%)	13 (52.0%)	0.989^c^
Hyperlipidemia	10 (62.5%)	12 (57.1%)	14 (56.0%)	0.914^c^
Cardiovascular diseases	6 (37.5%)	7 (33.3%)	9 (36.0%)	0.964^c^

a One-way ANOVA test,

b Mann–Whitney U test,

c chi-square test,

NDI = Neck Disability Index. Value in bold indicates statistical significance (p < 0.05).

**Table 2:** Postural stability scores by groups.

Surface and condition	Group Ia (n = 16)	Group Ib (n = 21)	Control group (n = 25)	p*	H(2)	η^2^	Pairwise comparison
**Firm surface**							
Eyes open	90.36 (71.86–96.69) [87.64–95.04]	91.60 (81.10–96.04) [90.05–95.15]	92.90 (74.56–95.92) [90.20–93.99]	0.628	0.932	0.000	–
Eyes closed	87.22 (58.20–92.28) [83.64–91.45]	90.54 (43.58–97.25) [83.97–93.23]	90.38 (81.13–96.42) [87.74–93.43]	0.047	6.135	0.052	1.000x; 0.046y; 0.197z
**Foam surface**							
Eyes open	83.27 (62.54–96.27) [75.98–90.66]	87.78 (75.12–92.31) [84.48–89.90]	89.44 (66.67–91.49) [85.06–91.49]	0.133	4.029	0.033	–
Eyes closed	76.19 (65.26–84.31) [72.06–82.31]	84.20 (67.55–93.50) [79.09–90.67]	87.25 (46.91–92.52) [81.51–90.33]	<0.001	16.906	0.115	1.000x; <0.001y; 0.005z

* Kruskal–Wallis test,

x: Group Ib - Control Group, y: Group Ia - Control Group, z: Group Ia -Group Ib.

## Footnotes

1 World Health Organization (2024). Falls [online]. Website https://www.who.int/news-room/fact-sheets/detail/falls [accessed 11 December 2025].
